# Qualitative exploration of 3D printing in Swedish healthcare: perceived effects and barriers

**DOI:** 10.1186/s12913-024-11975-0

**Published:** 2024-11-23

**Authors:** Olivya Marben Sag, Xiang Li, Beatrice Åman, Andreas Thor, Anders Brantnell

**Affiliations:** 1 Department of Surgical Sciences, Plastic & Oral and Maxillofacial Surgery, Uppsala University, Uppsala, 751 85 Sweden; 2 Department of Civil and Industrial Engineering, Industrial Engineering and Management, Uppsala University, Ångströmlaboratoriet, Lägerhyddsvägen 1, Uppsala, 752 37 Sweden; 3 Department of Women’s and Children’s Health, Healthcare Sciences and E-Health, Uppsala University, MTC-Huset, Dag Hammarskjölds Väg 14B, 1 Tr, Uppsala, 752 37 Sweden

**Keywords:** 3D printing, Healthcare, Adoption, Barriers, Effect, Qualitative

## Abstract

**Background:**

Three-dimensional (3D) printing produces objects by adding layers of material rather than mechanically reducing material. This production technology has several advantages and has been used in various medical fields to, for instance, improve the planning of complicated operations, customize medical devices, and enhance medical education. However, few existing studies focus on the adoption and the aspects that could influence or hinder the adoption of 3D printing.

**Objective:**

To describe the state of 3D printing in Sweden, explore the perceived effects of using 3D printing, and identify barriers to its adoption.

**Methods:**

A qualitative study with respondents from seven life science regions (i.e., healthcare regions with university hospitals) in Sweden. Semi-structured interviews were employed, involving 19 interviews, including one group interview. The respondents were key informants in terms of 3D printing adoption. Data collection occurred between April and May 2022 and then between February and May 2023. Thematic analysis was applied to identify patterns and themes.

**Results:**

All seven regions in Sweden used 3D printing, but none had an official adoption strategy. The most common applications were surgical planning and guides in clinical areas such as dentistry, orthopedics, and oral and maxillofacial surgery. Perceived effects of 3D printing included improved surgery, innovation, resource efficiency, and educational benefits. Barriers to adoption were categorized into organization, environment, and technology. Organizational barriers, such as high costs and lack of central decisions, were most prominent. Environmental barriers included a complex regulatory framework, uncertainty, and difficulty in interpreting regulations. Technological barriers were less frequent.

**Conclusions:**

The study highlights the widespread use of 3D printing in Swedish healthcare, primarily in surgical planning. Perceived benefits included improved surgical precision, innovation, resource efficiency, and educational enhancements. Barriers, especially organizational and regulatory challenges, play a significant role in hindering widespread adoption. Policymakers need comprehensive guidance on 3D printing adoption, considering the expensive nature of technology investments. Future studies could explore adoption in specific clinical fields and investigate adoption in non-life science regions within and outside Sweden.

**Supplementary Information:**

The online version contains supplementary material available at 10.1186/s12913-024-11975-0.

## Background.

Additive manufacturing (AM) or 3D printing (3DP) produces objects by adding layers of material instead of mechanically reducing material [1]. This production technology has several advantages such as rapid production of complex forms, use of materials with new capabilities, diminished use of material and lower transportation costs [2]. 3DP has been used in a variety of medical fields, including cardiology [3], various cancer forms [4], oral and maxillofacial surgery [5], orthopedics [6], and radiology [7]. 3DP in healthcare can have impact on healthcare and patient outcomes through several pathways: (1) to improve planning of complicated operations such as in cardiology [8], (2) to customize medical devices such as prothesis and implants [9], (3) to improve medical education [10], (4) to improve information to patients preceding surgery [11], (5) to produce, for instance, tissue and skin through bioprinting [12, 13], and (6) to tailor size and dosage of medication for patient’s needs [14]. To further illustrate the use of 3D printing in healthcare, exploring its adoption in oral and maxillofacial surgery provides valuable insights. A recent survey of oral and maxillofacial surgeons in Sweden revealed that 3DP is predominantly used in orthognathic and trauma surgeries, with anatomical models and surgical guides being the most common applications [15]. Two 3DP techniques—material extrusion and vat polymerization—were frequently employed, both of which are commonly used to print plastics [16]. Regarding software usage, many surgeons lacked detailed knowledge; however, those familiar with 3DP software reported using professional slicer programs such as Meshmixer and GrabCAD [15]. These types of slicer programs are commonly used to convert computer-aided design (CAD) or intraoral scan images into printable 3D models [17]. A separate survey of German oral and maxillofacial surgeons found that 3DP was mainly used in implantology, microvascular bone reconstruction, and orthognathic surgery, with anatomical models and drilling guides being the most frequently employed tools [18]. In summary, while 3D printing holds significant potential in various clinical sub-specialties of oral and maxillofacial surgery, its current application remains largely focused on simpler tasks, such as creating surgical guides.

Given the possible impact of 3DP in healthcare, Grinin et al. [1] picture 3DP and medical technologies as two of the drivers of cybernetic revolution leading to a phase of self-regulating systems in production. Despite the promises of 3DP in healthcare, there are barriers to adoption relating to the technology itself such as the high investment cost [19] or certain trade-offs with different printing technologies [20], relating to the society such as the complex regulatory framework [21], or relating to patient acceptability [22]. However, existing studies on 3DP in healthcare do not often focus on adoption although sometimes reporting on barriers to adoption, and thus it is likely that additional barriers to adoption exist such as surgeons interest and skills, existing organizational routines and organizational support as indicated by a qualitative study on adoption of 3DP in cardiology [23]. Further, very few studies have tried to capture 3DP adoption in an entire hospital or at a country level [15, 24]. Given this lack of studies on clinical adoption and country wide approaches policymakers do not have a comprehensive guidance concerning adoption of 3DP. Investing in new technology such as 3DP is often expensive [19], and for policymakers the perceived effect is an important aspect that could influence decisions to invest in 3DP. Perceived barriers to adoption in turn can reveal the possible factors hindering adoption, and thus something that policymakers need to be aware of when investing in 3DP. To this end, this study has three objectives: (1) to describe the state of affairs of 3D printing in Sweden, (2) explore the perceived effects of using 3D printing and (3) identify the barriers to adoption of 3D printing.

## Methods

### Study design and setting

A qualitative study including respondents from seven life science regions responsible for healthcare provision in Sweden was conducted. While reporting the findings the Standards for Reporting Qualitative Research (SRQR) guidelines were adhered to [25]. The focus of this study was on the Swedish healthcare system and adoption of 3DP among the seven life science regions. Sweden is divided into 21 regions responsible for healthcare. Out of these, seven regions have medical universities including university hospitals. To this end, these regions are labelled as life science regions. The life science regions are an important starting point to understand adoption of 3DP since the life science regions are pictured as the engines of life science innovation in Sweden [26]. Many of them are large regions but there are also medium and small regions in terms of population size. In addition, they provide a good geographical distribution covering north, south, west and east of Sweden (for details on life science regions see Table 1).

**Table 1:** Key characteristics of the seven life science regions in Sweden

**Region**	**Number of hospitals in the region**	**Population in the region**	**Geographical location**
1	Seven hospitals, two of them university hospitals	2,450,921 (largest in the country)	East Svealand
2	Eight in total, one of them a university hospital	1,744, 859 (second largest in the country)	Western coast of Sweden
3	10 in total, two of them university hospitals	1,402,425 (third largest in the country)	Southernmost region of Sweden
4	Three in total, one of them a university hospital	469,704 (fourth largest in the country)	Southeastern of Sweden
5	Two in total, one of them a university hospital	395, 026 (fifth largest in the country)	Eastern coast of Sweden
6	Three in total, one of them a university hospital	308,007(8th largest in the country)	South Svealand
7	Three hospitals, one of them a university hospital	276,545(14th largest in the country)	North-east of Sweden

### Respondents

Based on our previous experience and work with 3DP we knew that each of the regions did not have a contact person for 3DP adoption or similar positions, rather the responsibility for and interest towards 3DP was dispersed between different roles and professions. To identify key people around 3DP, we employed several strategies (1) we searched the hospital and regional websites for news and information about 3DP and reached out to people mentioned in these, (2) we reached out to people that we already knew worked with 3DP in the target regions, (3) we contacted the units for medical technology at hospitals, (4) we contacted people at 3DP labs within the regions, (5) we reached out to all business developers within these regions, and (6) we talked to researchers, innovation support agencies and industry people to identify key professionals. Initial contact was made via email, followed by phone calls when possible and necessary. Based on this, we ended up with a mixed group of key professionals (*n* = 17) in each of the seven regions including engineers, medical professionals, business developers and managers (see Table 2). The regions and respondents are anonymized to protect the privacy of the respondents, and thus the regions will be labelled as region A, B, C in the continuation.

**Table 2:** Key professionals working with 3DP in the Swedish life science regions

Respondent	Region	Position in the region	Gender	Number of interviews
R1RA	A	Head of department (Medtech R&D)	Male	1
R2RA	A	Dentist	Male	1
R1RB	B	Engineer	Female	2
R2RB	B	Medical technology safety strategist	Male	1
R3RB	B	Regional developer	Female	1
R1RC	C	Medical doctor (PhD student)	Female	1
R2RC	C	Deputy chief pharmacist	Male	1
R3RC	C	Collaboration leader	Female	1
R4RC	C	Business strategist	Male	1
R5RC	C	Project Manager	Male	1
R1RD	D	Medical director	Male	1
R1RE	E	Medical engineer	Male	1
R1RF	F	Business strategist	Male	1
R2RF	F	Business investigator	Male	1
R3RF	F	Operation manager	Female	1
R1RG	G	Surgeon (oral and maxillofacial)	Male	1
R2RG	G	Engineer	Male	2

### Data collection

To capture the different viewpoints but still be able to compare the findings we employed semi-structured interviews (*n* = 19) of which one interview was a group interview (with region F). The majority of the interviews were conducted online, through the Zoom online platform, due to the busy schedule of the respondents. Data collection took place in two occasions, first between April and May in 2022 and then between February and May 2023. During the first occasion, we were not able to cover all seven regions and thus we complemented with interviews during the second occasion. In addition, two respondents were very busy during the initial interview, so we were unable to cover all the questions. As a result, they were re-interviewed during the second occasion (for details on interviews see Table 2). The interview guides were modified between the two rounds based on insights gained from the first round. We found that the initial guide contained too many questions, which could overwhelm respondents. Additionally, we realized that reorganizing the order of questions could enhance the flow of the interview. In the second round, we aimed to collect more information about the respondents; however, we decided not to include this data in our analysis. We also improved the wording in the second version for better clarity and engagement. Despite these adjustments, the core topics—adoption characteristics, effects, and barriers—remained consistent in both rounds, making it feasible to combine the data from both interview sessions (See Supplementary file 1 for the English versions of the interview guides). The interview guides were developed based on existing research. Both interview guides were pilot tested and adjusted based on the outcomes. The interviews were conducted in either English or Swedish, depending on the language proficiency of the interviewer. The interviews lasted between 30 and 60 min. The interviews were recorded and transcribed. We continued conducting interviews until data saturation was reached across all regions, meaning no new information emerged. In evaluating data saturation, we focused on the themes and subthemes arising from the data, considering saturation achieved when no new insights were added to these in subsequent interviews. We did not emphasize professional backgrounds or individual regions during this process, as the respondents’ professional backgrounds were not homogeneous and the number of participants from each region varied.

### Data analysis

For the first objective, we conducted a descriptive analysis and presented the findings in a table. For the second and third objective, we conducted an inductive thematic analysis of the interview data [27]. Inductive analysis aims to capture the perspectives and knowledge of respondents without imposing predefined expectations on what is important in the data, in contrast to a deductive approach [28]. This openness and flexibility are particularly valuable when the phenomena—specifically, perceived effects of using 3DP and barriers to adoption—are not well explored in existing research, and few established frameworks exist to guide the analysis [29]. For these reasons, an inductive thematic analysis was employed. We divided the analysis into six steps. First, we coded the interviews based on the objectives. Second, we compared the codes across the interviews and merged similar codes. Third, we identified similarities and differences between the codes and divided these under initial subthemes. Fourth, based on the subthemes we identified a common higher-level theme for similar subthemes and placed the subthemes under these. Fifth, we developed clear labels for the themes and the subthemes. Sixth, we identified all supporting quotes for the subthemes and organized these into a table. This procedure also allowed us to identify the most common subthemes raised by the respondents. Seventh, we presented the themes, subthemes and supporting quotes in a table (Supplementary file 2 and 3). However, for the third objective, we also connected the findings with an existing framework to study innovation adoption, the Technology-Organization-Environment (TOE) framework [30], and divided the developed subthemes under technology, organization and environment. The preliminary coding was done by one researcher and independently validated by another researcher. The final coding was discussed in the research group consisting of a dentist with a specialization in oral and maxillofacial surgery with experience of 3DP, a dentist, and a researcher with experience of 3DP adoption. The findings were sent to all respondent for feedback to increase validity of the findings [31]. All respondents were satisfied, and no suggestions for improvement were received.

## Results

### State of affairs concerning 3DP in the Swedish life science regions

Altogether 19 interviews were conducted with 17 respondents identified as the key professionals working with 3DP in the seven life science regions in Sweden. All seven regions worked with 3DP but none of them had an official strategy for adoption of 3DP. Majority of the hospitals had access to both inhouse produced and externally supplied 3DP applications. The most common 3D printed applications were surgical planning applications and surgical guides. Further, between three and eight clinical areas used 3DP such as dentistry, orthopedics, maxillofacial surgery, neurosurgery and hand surgery (for details, see Table 3).

**Table 3:** Key characteristics for the seven life science regions concerning 3DP adoption

Region	Works with 3DP in healthcare (Yes/No)	Applications for 3DP in healthcare?	Clinical areas using 3DP?	Access to 3DP (inhouse vs. external)	Official strategy for 3DP in healthcare? (Yes/No)
A	Yes	-Surgical planning -Surgical guides -Nerv damage models -Cellular therapy -Teaching models -Dentures	-Dentistry -Maxillofacial surgery -Orthopedics -Neurosurgery	Both	No
B	Yes	-Facial prosthetics -Hand surgery -Bite guards in dentistry -Cellular therapy -Pedagogical models -Presurgical planning -Visualization -Anatomical models -Surgical guides -Patient specific tools	-Dentistry -Orthopedics -Cardiology -Maxillofacial surgery -Radiation therapy -Hand surgery	Both	No
C	Yes	-Drugs -Models for surgical planning -Surgical guides -Patient specific implants -Bio Printing -Orthopedical implants -Teaching models -Joint protheses -Communication method between neurosurgeons and other specialties	-Drug industry -Dentistry -Maxillofacial surgery -Orthopedics -Neurosurgery -Hand surgery -Radiology -Bioprinting	Extern	No
D	Yes	-Vessel and ear, nose, and throat prints -Skeleton prints -Surgical template -Anatomical prints	-Ear, nose and throat -Radiology -Surgery -Orthopedics	Both	No
E	Yes	-Surgical guides -Anatomical prints for surgical planning -Spine surgery -Hand surgery	-Orthopedics -Plastic surgery -Spine surgery -Hand surgery -Maxillofacial surgery	In-house	No
F	Yes	-Orthopedic clinical models -Surgical planning -Jaw reconstruction planning	-Orthopedics -Maxillofacial surgery -oral surgery	Both	No
G	Yes	-Molding patterns for implants -Wafers -Anatomical models -Surgical templates	-Plastic surgery -Maxillofacial surgery -Neurosurgery -Cardiology -Orthopedics -Malignancy treatment	Both	No

The remaining aspects pertaining to the two objectives are discussed below with illustrative quotes. We will start by discussing the perceived effects of using 3DP and continue with the perceived barriers to adopting 3DP.

### Perceived effects of using 3DP in healthcare

The respondents identified various effects of using 3DP distributed along four broad themes: (1) improved surgery, (2) innovation and development, (3) improved use of resources and (4) education. For details on all themes and subthemes see Supplementary file 2.

### Improved surgery

Improved surgery (*n* = 15) was the most frequent theme consisting of four subthemes: surgical training and planning (*n* = 7), improved surgical precision (*n* = 4), reduced risk of complications (*n* = 4) and surgical guides (*n* = 3). Improved surgery was mentioned by at least one respondent from each of the seven regions. Respondents highlighted the improved surgical precision when using 3DP thanks to surgical guides and custom tools used during surgery, guiding the surgeons in placing correct cut and thereafter the entire surgical procedure:

*“* The effect and advantage of it [*using 3DP*] is that surgery and the desired result [*the outcome*] can be more precise.*” (R2RA).* The surgical guides were utilized in different clinical areas, such as orthopedics, maxillofacial surgery, hand surgery and dentistry. Further, presurgical 3D-planning was identified as a factor reducing surgical complications. The reduction is presented down to half in one clinical area*:” But on the other hand, for example on cranioplasty, where our preliminary data indicate that we have reduced our complication rate almost to half.” (R2RG).* 3D planning was also perceived to benefit training for surgeons as stated by one respondent: *However, with 3D printing and planning tools, surgeons can better visualize procedures, spot potential errors, and even test surgeries virtually. This technology enhances the surgical process by offering a more detailed and effective planning approach.” (R2RB).*

### Innovation and development

Innovation and development (*n* = 8) was the second most frequent theme consisting of two subthemes: development of tailored applications (*n* = 4), and possibility to offer tailored medicines (*n* = 4). Innovation and development was raised by four regions. The respondents acknowledged that 3DP aligns well with the general trend of making healthcare more personalized: *“The individuality these technologies offer can be highly beneficial. Healthcare is becoming more personalized, and this technology aligns with that trend.” (R2RF).* Further, one region stated that they are working to develop 3DP of individualized medicines in house for children with severe cancer or neurological diseases, even though it was still on the lab level. None of the other regions mentioned 3DP of medicines.

### Improved use of resources

Improved use of resources (*n* = 7) was the third most frequent theme consisting of two subthemes: time efficiency (*n* = 4), and reduced costs (*n* = 3). Improved use of resources was raised by four regions. The respondents agreed on that using 3DP improves efficiency of surgery: *“And then, the big advantage, where it gets really interesting, at least in a Swedish healthcare context, is that you can come to the conclusion that you can squeeze another operation into the day.” (R2RG).* The respondents that raised the cost aspects considered that 3DP is cost-effective and that in the long run it will reduce costs: *“Furthermore, the manufacturing method also provides more efficient care, lower costs in the long term as you usually do not have to redo the operation, for example.” (R3RC).*

### Education

Education (*n* = 4) was the least frequent theme consisting of one subtheme: demonstration and teaching patients (*n* = 4). Education was raised by four regions. The respondents agreed on that using 3DP improves communication with patients, for instance pre-surgery. Showing a 3D printed model can aid patients to understand the procedure: *“We need to include more educational aspects. Sometimes we print out a model to be able to explain to patients…”(R3RB).*

### Barries to adoption of 3DP

The barriers influencing adoption of 3DP identified by the respondents were divided on three broad themes based on the TOE framework: (1) technology, (2) organization, and (3) environment. Within these themes we identified a set of subthemes that are presented below starting with the most frequent theme. For details on all themes and subthemes see Supplementary file 3.

### Organization

Organization (*n* = 33) was the most frequent theme consisting of seven subthemes: costs (*n* = 7), no central decision at the hospital level (*n* = 6), requirements for premises (*n* = 4), requirements for support (*n* = 4), requirements for technical competence (*n* = 4), unclear need (*n* = 4), and conservative healthcare professionals (*n* = 4). Costs is the most highlighted barrier by seven respondents from six regions. Regulatory process, printers and printer related components and materials such as metals used in printers require high investments: “*It’s the costs. That the printer is expensive, that the material is expensive.” (R1RD).* Lack of central decision at the hospital level is another point of concern. Respondents from six regions acknowledged that their hospital does not have a strategy for implementation of 3DP. The technology is commonly adopted by individual doctors who have interest for or identified the need of 3DP: *“Who introduced it? Doctors, hospitals, or patient demand? So far, it’s the doctors. They’ve been driving this due to their enthusiasm. Decisions are often made at different levels within the decentralized healthcare organization…” (R2RF).* Further, finding suitable premises that meet different requirements such as handling toxic gases and substances seems to be challenging. Requirements for support and technical competence are other frequently identified barriers:” *The 3D printing technology requires special knowledge and skills, which makes it difficult for some companies or hospitals to introduce the technology…”( R4RC).* Further the need for the technology is unclear for some. For instance, a medical director from one region indicates that only one 3D-printed model is requested per month. The low request of the technology is confirmed by three other regions. Further, the technology is met by conservatism, especially by “old-school” surgeons who are skeptical about the effectiveness of 3D planning:*”There may be some conservatism in healthcare when it comes to embracing new technologies like 3D printing. It could be hard to get people to consider adopting the technology if they can’t see clear benefits and cost savings.” (R2RA).*

### Environment

Environment (*n* = 15) was the second most frequent theme consisting of three subthemes: complex regulatory framework (*n* = 7), uncertainty about regulations (*n* = 5) and difficult to interpret regulations (*n* = 3). Complex regulatory framework was raised as a barrier by respondents from five regions. Regulations are described as strict in terms of requirement for different approvals for different production parts of 3DP, which is time consuming and requires high investments. Further the difference in requirements for producing different materials falls under different categories in the regulation: “*A current barrier is the MDR regulations. The reason behind this is the increased processing requirements.”( R1RE).* There is uncertainty about regulations in regions, leading individuals to either work with 3DP with limited considerations to regulation or limit their work to lowest production levels possible, due to fear for doing wrong: “*Yes, I mean, MDR has been a mess to deal with because of that. So, it hasn’t exactly made our job any easier. But I will say this, you have to have somebody who is quite regulatory savvy, and that’s hard to find, I’ll say. It’s easier to find a 3D printing geek, but finding an MDR geek is more difficult, and in my case it’s been me who has had to read and learn with MDR. So that it is a challenge*.*”(R2RG).* Further, respondents claim that regulations are hard to interpret, leaving room for different interpretations between regions and sometimes even within regions. Interpretation difficulties concern for instance methods, application approach and patient safety: “*In addition, the interpretation of the law depends on each region. It’s not just a law that you learn, but each region interprets it in its own way, which makes it even more difficult. But if region help each other and exchange regulation knowledge under the MDR, I think it would be very valuable. It would be a success.”(R1RF, R2RF, R3RF).*

### Technology

Technology (*n* = 4) was the least frequent theme consisting of one subtheme: not applicable for all areas (*n* = 4). Respondents from three different regions mentioned that the 3DP technology is not applicable in all health care areas. For instance, it depicts the skeleton more precisely than soft tissue and narrow blood vessels. Further, 3D-printed surgical guides are not always trustable in terms of precision and might complicate the surgery compared to traditional methods in some cases: “*For instance, if a surgeon is experienced in fixing a hand fracture, they may not see the need for a 3D plan. But in unique cases, they may not be aware that they can contact us for 3D planning.”(R1RE).*

## Discussion

In this study, our aim was to describe the state of 3DP in Sweden, explore the perceived effects of using 3DP, and identify barriers to its adoption. Below, we will highlight the three key findings from our study.

### Widespread 3DP adoption with no formal strategy

The study revealed that all seven life science regions in Sweden actively used 3DP in healthcare, particularly in surgical planning and employing surgical guides across various clinical areas such as in oral and maxillofacial surgery and orthopedics, which is in line with existing findings on 3DP adoption in a Danish hospital [24]. Although wide spread adoption, the findings show that the use is still centered around less advanced use of 3DP whereas existing research depicts more advanced use of 3DP in oral and maxillofacial surgery such as patient specific implants and total joint replacements [32]. In contrast, existing research on adoption of 3DP in orthopedics indicate the use of less advanced applications of 3DP such as surgical planning and patient education [33], which are in line with our findings. Still, few studies exist that would have studied actual adoption of 3DP. According to our understanding none of the existing studies on 3DP adoption have taken a broader perspective covering several clinical fields and contexts for instance a national or regional approach. Existing studies from Sweden and Germany on adoption of 3DP in oral and maxillofacial surgery revealed large scale adoption (around 60% use rate) but with use focused on less advanced applications [15, 18]. Despite this widespread use among the studied life science regions in this study, a notable finding was the absence of an official adoption strategy in all regions. The lack of a structured approach indicates that the adoption of 3DP has largely been driven by individual initiatives rather than a comprehensive, coordinated effort at the organizational level. Existing approaches to plan for change and develop change strategies underscore the importance of bottom-up initiates (needs-based strategies) but also highlight the importance of involving top leadership and key decision makers in organizational change initiatives to ensure successful outcomes [34, 35]. Research shows that involving top leadership and key decision makers can ensure funding and organizational support for the new technology, and result in integration of the technology in the organizational functioning [34]. To this end, the study findings underscore the need to involve top leadership and key decision makers in each of the regions to harness the full potential of 3DP in healthcare. Although this study primarily focuses on 3D printing in healthcare, this technological shift is part of a broader trend toward digitalization in the healthcare sector. Given that 3D printing relies on digital applications, the findings related to strategy, funding, and decision-maker involvement are likely applicable to other aspects of healthcare digitalization, including initiatives involving artificial intelligence, the Internet of Things, and blockchain [36–38].

### Several positive perceived effects on healthcare

Respondents identified several positive effects of 3DP in healthcare, encompassing improved surgery, innovation, resource efficiency, and educational benefits. Improved surgical precision, guided by 3D-printed models, emerged as a prominent theme. This finding aligns with existing research on dentistry emphasizing the utility of 3DP in enhancing surgical outcomes [5, 19]. The perceived benefits also extended to innovation and development, with the potential for personalized applications and even individualized medicines. Personalized applications such as patient specific implants are often discussed and explored in existing research, and also raised as some of the benefits of 3DP [32]. Likewise, personalized medicines tailored for specific patient characteristics such as age, weight and disease state are frequently explored in existing research [21, 22]. Additionally, the technology was seen to improve resource efficiency, both in terms of time and cost, indicating its potential economic advantages. Interestingly, existing research is inconclusive regarding the cost and cost-effectiveness of 3DP. One review focusing on dentistry found out that starting costs are relatively high in terms of purchasing printers and training clinicians [19]. Another review on dentistry found that using 3DP was somewhat cost-effective, in detail, although there were high investment costs in the beginning the work flow entailed less involvement of clinicians balancing out the costs [5]. Still, very few studies have covered the actual costs of adopting and using 3DP. One small scale evaluation of complete dentures compared the costs of using conventional and digital dentures, and stated that using digital dentures entailed a lower total cost [39]. Another study focusing on production of dentures in a large clinic in US evaluated the cost of using digital (3D printed) dentures, and found out that digital dentures entailed a significant cost saving [40]. To understand better how 3DP could lower or influence the costs in the studied life science regions in clinical fields such as oral and maxillofacial surgery and orthopedics a further in-depth investigation is necessary including stakeholders at different levels in the organization. To summarize, the study findings on perceived effects on healthcare are largely supported in existing research. However, some of these perceived effects are not yet reality such as 3DP of tailored medicines.

### Organizational and regulatory barriers to adoption

Despite the numerous benefits of 3DP, significant barriers to its adoption were identified, with organizational challenges taking precedence. The study highlights high costs associated with 3DP technology and the absence of centralized decision-making at the hospital level as major obstacles. This decentralized approach, driven by individual enthusiasts rather than institutional strategy, poses challenges to sustained and coordinated adoption, including difficulties in securing funding. While existing research on the cost of 3DP remains inadequate, focusing primarily on specific areas such as denture production, it is evident that investment costs for purchasing printers and training clinical staff entail considerable expenses [19]. In hospital settings with tightly controlled budgets, a central decision to invest becomes imperative. When top leadership and key decision makers are not involved, a lack of central strategy and funding often ensues. Given the central role of cost-effectiveness in healthcare provision and the need for budgetary vigilance [41, 42], more comprehensive studies on the starting and operating costs of 3DP are needed. Another interesting finding regarding organizational barriers is the conservatism among clinicians, some of whom are reluctant to adopt new technologies. In innovation adoption literature, it is established that attention should be given to adopters, notably early and adopters and early majority, rather than laggards (i.e., conservatives) [43]. While early adopters are often receptive, persuading the early majority, essential for market success, is more challenging [44]. Consequently, identifying adopter categories and devising suitable implementation strategies are critical challenges for life science regions. Future studies demonstrating cost-effectiveness and improved outcomes may sway the early majority but not laggards.

Besides organizational barriers, the regulatory environment poses a significant hurdle to the adoption of 3DP. The new EU Medical Device Regulation (MDR) was perceived to complicate matters, hindering adoption. This aligns with existing understanding of the MDR’s impact on 3DP [45], reflecting the historical tension between medical device regulations and 3DP [46]. Interestingly, the FDA in the US appears more proactive in facilitating 3DP development and adoption compared to the EU’s MDR [47]. A recent study on the impact of MDR on 3D printing in the European context highlights several challenges related to the regulatory framework [45]. These include the need for a risk and quality management system to oversee 3DP production in hospitals, training personnel on both the risk management system and technical aspects, and evaluating whether internal certification is required for materials and software [45]. Similarly, a study in the USA identified regulatory hurdles concerning Food and Drug Administation (FDA) regulations on 3DP [46]. It found that the customizability of 3DP solutions complicates the drafting of a design control model for market approval, while the unique production processes pose challenges to the quality assurance methods originally based on mass production. Additionally, the changing properties of material powders make it difficult to meet FDA regulatory standards [46]. Overcoming regulatory barriers requires access to qualified professionals with regulatory expertise, which is a common challenge across EU countries due to the need to interpret the revised regulations and their impact on users [48]. Furthermore, establishing standardization and developing clinical standards for 3DP could help address some of the regulatory issues [45].

### Limitations

The interviews were conducted with participants who had different educational backgrounds, work experiences, and roles. Additionally, there were varying numbers of respondents from each region. In some instances, two interviews were conducted with the same respondent, while in other cases, one interview was held with three respondents together. This variability might have influenced uniformity and transparency. In general, the study’s low sample size may affect its overall significance. However, our findings indicate that 3DP adoption in the seven life science regions was largely driven by a small group of individuals, making it challenging to involve a larger number of key stakeholders. Furthermore, respondents’ perceptions could be partly dependent on their work roles. The presence of six participants from one region and one from another might have impacted the details provided about the region’s involvement with 3DP. Nevertheless, our aim was to achieve saturation among the respondents, indicating that the identified perceptions regarding effects and barriers are representative of all seven regions. Another limitation is that we adjusted the interview questions between the two cohorts, which could introduce some variability in the responses. However, since the essence of the core questions remained consistent in both instances, the risk of significant variability should be minimal. Due to the bottom-up approach in the implementation of 3DP across the seven regions, it is challenging to pinpoint key respondents driving the implementation. A future study could stem from the clinical fields identified in this study and adopt a bottom-up approach to studying the implementation of 3DP. Additionally, it would be interesting to explore whether the state of affairs is similar in healthcare regions that are not perceived as engines of medical development, specifically non-life science healthcare regions, both within Sweden and beyond.

## Conclusion

There are several effects and barriers that influence the implementation and use of 3DP in health care in Sweden. The identified perceived effects of use were related to improved surgery, development and innovation, improved use of resources and educational purposes. The barriers were associated with organization, environment, and technology, with organizational and regulatory barriers appearing to play a more significant role in slowing down the further implementation of 3DP in Sweden. Policymakers need comprehensive guidance on 3D printing adoption, particularly given the significant investment costs involved. Developing formal national or regional strategies for 3DP could help unlock its full potential. Involving top leadership and key decision-makers would be crucial to securing funding and ensuring a coordinated rollout, enabling broader and more advanced applications. Future studies should focus on 3DP adoption in specific clinical fields and explore its implementation in non-life science regions, both within and beyond Sweden, to further inform healthcare management and policymaking.

## Supplementary Information

Supplementary Material 1.

Supplementary Material 2.

Supplementary Material 3.

## Data Availability

All data generated or analysed during this study are included within the article and its additional files.

## Abbreviations

AM: Additive Manufacturing
3DP: 3D printing
SRQR: Standards for Reporting Qualitative Research
MDR: Medical Device Regulation
