# Supplementary material for: Qualitative exploration of 3D printing in Swedish healthcare: perceived effects and barriers
Source: BMC Health Serv Res. 2024 Nov 23;24:1455. doi: 10.1186/s12913-024-11975-0 (PMC11585134; doi:10.1186/s12913-024-11975-0)
Supplement: Supplementary file 1 — Supplementary Material 1. [file 12913_2024_11975_MOESM1_ESM.docx]

**Supplementary file 1: Interview guide AM adoption in regions**

**Interview guide (April and May in 2022 – Interviews conducted in English)**

1. Would you like to introduce yourself briefly? (Name, age, occupation, organization) (ask all)
2. Could you briefly describe your work? (ask all)
3. Are you aware of 3D printing/Additive Manufacturing (AM)? (ask all)
4. What is your perspective on additive manufacturing in healthcare? (ask all)
5. Is your region working with AM in healthcare? (ask all)
6. What is your role in these projects or initiatives? Can you provide examples of these projects or strategies? (ask those who work with AM)
7. Which clinical areas have started to adopt AM? (ask those who work with AM)
8. Why is AM being adopted in healthcare? (ask those who work with AM)
9. Who decided to adopt AM in healthcare? (ask those who work with AM)
10. How was the decision made to adopt AM? Was there a pilot project? (ask those who work with AM)
11. What do you think about the effects of using AM? (ask all)
12. What changes are needed to adopt AM in healthcare? (ask all)
13. How complicated is it to adopt AM in healthcare? Can you identify any hindrances? (ask all)
14. Does the adoption of AM entail costs? How have the costs impacted the decision to adopt AM? (ask all)
15. What advantages does AM have in healthcare compared to traditional manufacturing? And for whom? (ask all)
16. What disadvantages does AM have in healthcare compared to traditional manufacturing? And for whom? (ask all)
17. How are patients treated with AM? (ask all)
18. How do patients benefit from AM in healthcare compared to traditional technology? (ask all)
19. Are patients interested in AM? (ask all)
20. What kind of technical problems does AM adoption bring? How are these problems resolved? (ask all)
21. Are doctors and medical professionals trained to work with AM? (ask all)
22. Can hospitals manage to design and produce AM-related devices on their own (in-house)? If not, how does your organization get access to 3D-printed parts? How does the supply chain look? (ask all)
23. What do you think about other regions' use of AM? Are there any meetings or communications regarding experience sharing or opinion exchange? (ask all)
24. What kind of local or state policies and regulations influence the adoption of AM? How do they influence adoption? What is needed in terms of policies and regulations to facilitate adoption? (ask all)

**Interview guide (February and May 2023 – Interviews conducted in Swedish)**

1. What is your name and which organization do you work for? (ask all)
2. What is your academic and professional background? (ask all)
3. Could you briefly describe your work? (ask all)
4. What kind of knowledge do you have about 3D printing/additive manufacturing? (ask all)
5. What is your perspective on additive manufacturing (AM) in healthcare? (ask all)
6. Is your region working with AM in healthcare? (ask all)
7. How is your region working with AM in healthcare? Can you provide examples? (ask those who work with AM)
8. What is your role in this work? (ask those who work with AM)
9. Does your region have a strategy for working with AM? In which document is this described? Can you send me the document? (ask all)
10. Which clinical areas have started to adopt AM? (ask those who work with AM)
11. Why is AM being adopted in healthcare in your region? (ask those who work with AM)
12. Who decided to adopt AM in healthcare in your region? (ask those who work with AM)
13. How was the decision made to adopt AM in healthcare in your region? (ask those who work with AM)
14. What do you think about the effects of using AM in healthcare? (ask all)
15. Are there any barriers to adoption that you can identify in your region? (ask all)
16. What advantages does AM have in healthcare compared to traditional manufacturing? And for whom? (ask all)
17. How does your region obtain AM-printed solutions? (in-house vs. externally purchased) Is this arrangement optimal? Why? (ask those who work with AM)
18. What do you think about other regions' use of AM? (ask all)
19. Can you think of any other people in your region that I should talk to about AM adoption? (ask all)
