# Supplementary material for: Qualitative exploration of 3D printing in Swedish healthcare: perceived effects and barriers
Source: BMC Health Serv Res. 2024 Nov 23;24:1455. doi: 10.1186/s12913-024-11975-0 (PMC11585134; doi:10.1186/s12913-024-11975-0)
Supplement: Supplementary file 2 — Supplementary Material 2. [file 12913_2024_11975_MOESM2_ESM.docx]

| **Supplementary file 2. Perceived effects of using 3D printing among the seven life science regions** | | |
| --- | --- | --- |
| **Themes** | **Subthemes** | **Quotes** |
| **Improved surgery (n=18)** | Surgical training and planning (n=7) | “3DP models are used to train surgeons before operations, which reduces the risk of complications during the operation...”- R3RC |
|  |  | “…Radiology doctors work with imaging technologies, such as computer tomography (CT) scans, to create models for surgical planning or educational purposes.”- R4RC |
|  |  | ”A big thing is that you can be better prepared before the operation and then the operation will be safer. You feel safer as a surgeon, so to speak. So it makes sense that you then... if you have practiced through it, the surgery can go faster.”- R2RG |
|  |  | “However, with 3D printing and planning tools, surgeons can better visualize procedures, spot potential errors, and even test surgeries virtually. This technology enhances the surgical process by offering a more detailed and effective planning approach."- R2RB |
|  |  | “Improved surgical planning and understanding of anatomy…- R1RE |
|  |  | "It's more efficient for surgical planning. Integrating it into workflow and achieving efficiency can be a challenge, though."- R1RF, R2RF, R3RF |
|  |  | “In conclusion, the use and research of 3D printing in healthcare in this region has led to direct effects such as patient-adapted models for surgical planning and increased interest from doctors and surgeons.”- R6RC |
|  | Improved surgical precision (n=4) | ”The effect and advantage of it [using 3DP] is that surgery and the desired result [the outcome] can be more precise.”- R2RA |
|  |  | “By using 3D printing to manufacture prototypes of different fractures or medical structures, doctors and healthcare professionals can gain a better understanding of the patient's unique conditions and possible treatment options.”-R1RC |
|  |  | -“[S]urgical templates can lead to better results through precision and correct placement of incisions and surgical intervention.”- R1RD |
|  |  | "On the one hand, the technology created new surgical opportunities that did not exist before. It did also facilitate existing methods. It improved the quality of the surgery , since it’s reliable and more accurate now. But it did also help the process."- R1RG |
|  | Reduced risk of complications (n=4) | ”By using 3D planning, we can anticipate and address complications, leading to fewer post-surgical complications for the patient.”- R1RE |
|  |  | “Furthermore, good effects come from the fact that the patient-specific implants that can be manufactured with 3D-planning. The patient receives something that is customized for him and his surgery, which makes it easier for the body to heal, and the risk of complications after his surgery is reduced.”- R3RC |
|  |  | ”But on the other hand, for example on cranioplasty, where our preliminary data indicate that we have reduced our complication rate almost to half.”- R2RG |
|  |  | "Studies have shown that surgeons who are well-prepared and familiar with using the technology can decrease complications and improve efficiency. "- R2RB |
|  | Surgical guides (n=3) | “Custom tools and guides lead to better patient care, shorter surgeries, and informed consent from the patients. The evidence supporting these benefits is growing.”- R6RC |
|  |  | "[W]e have used 3D printing in many different ways. We have used it as guide material in connection with the surgery. Ee have also been able to print the guides in-house, i.e. at the hospital…"- R1RG |
|  |  | ”[S]urgical guides are good. The technology is used a lot among niche or general dentists in the private sector. Because then you use that technology….Your surgery is much safer.”- R2RA |
| **Innovation and development (n=8)** | Development of tailored applications (n=4) | "The individuality these technologies offer can be highly beneficial. Healthcare is becoming more personalized, and this technology aligns with that trend."- R2RF |
|  |  | “Through 3DP projects, the region can develop and implement new innovations in healthcare. Stimulation of innovation and development of new products and solutions.”- R1RA |
|  |  | “I see that it is a great opportunity and I believe that within a period of 5-10 years it will be a requirement to be able to deliver 3D printing, for example individually adapted prostheses.”- R1RF |
|  |  | "I think we will have a lot more 3D printed products in the future. There are incredible possibilities with it. You can get the right product for the right patient. But, I think we are an early start. And that we may have to experiment a bit.”- R3RB |
|  | Possibility to offer tailored medicines (n=4) | ”The project focuses on 3D printing of individually adapted medicines for seriously ill children where we have mainly focused on children with cancer or neurological diseases. And focus on formulation aspects when it comes to poorly soluble drugs and the use of lipid-based formulations for 3D printing.”- R1RC |
|  |  | “Enables the manufacture of medicines in-house with the correct dosage. Quick availability... Advantage with precision in dosage.”- R2RC |
|  |  | "Yes, there are efforts to make personal patient-specific medicine using AM. As of now, it's still mostly on the lab-based level, not entirely commercialized. It's a research-based cooperation between Tech and med farm faculties.”- R4RC |
|  |  | “When it comes to this pharmaceutical part, we have done a lot here in region 3. I don't know how long the development of the technology have reached in Sweden. After all, there are other regions in Europe where they have come much further with that and where they already print medicines in hospitals. However, there are projects and investments that are so far advanced that it is believed that the technology will be useful for in-house printing in short time.”- R3RC |
| **Improved use of resources** (n=7) | Time efficiency (n=4) | ”It is that you save both time and money... If you are going to have this in your own clinic and have collaboration with a few other clinics, then it is profitable.”- R2RA |
|  |  | “And then, the big advantage, where it gets really interesting, at least in a Swedish healthcare context, is that you can come to the conclusion that you can squeeze another operation into the day.”- R2RG |
|  |  | "I see that it is a great opportunity [...] you want shorter surgery time, you want to be efficient and  you want it to be as good as possible for the patient in order to get a better outcome.” - R1RF,R2RF,R3RF |
|  |  | "Personally, we believe the benefits outweigh the drawbacks. Clinical studies show that using 3D visualization enhances surgeon speed and reduces mistakes. It's more efficient for surgical planning. Integrating it into workflow and achieving efficiency can be a challenge, though."- R2RF,R3RF |
|  | Reduced costs (n=3) | “Furthermore, the manufacturing method also provides more efficient care, lower costs in the long term as you usually do not have to redo the operation, for example.”- R3RC |
|  |  | “Additive manufacturing is useful for these applications because it allows the production of custom components more efficiently and cost-effectively compared to traditional methods like milling.”- R6RC |
|  |  | ”Regarding cost, some discussions have arisen, but the general consensus is that the technology is cost-effective, particularly when considering factors like reduced re-infections.”- R2RG |
| **Education** (n=4) | Demonstration and teaching patients (n=4) | "There may be requirements for other parts, for instance teaching models that are used, showing and demonstrating for patients to increase understanding…”- R1RF,R2RF,R3RF |
|  |  | "3D models can aid patient understanding and informed consent, which can be beneficial.” - R6RC |
|  |  | “We need to include more educational aspects. Sometimes we print out a model to be able to explain to patients...”- R3RB |
|  |  | “We're conducting studies, and informed consent is often met positively. In cases where models help communicate with patients, such as children, they're appreciated. Patients have reported that it's helpful and sometimes emotionally stressful to see the models. However, it can also provide clarity and understanding, making it less about the model itself and more about the message it conveys.”- R2RG |
