# Supplementary material for: Qualitative exploration of 3D printing in Swedish healthcare: perceived effects and barriers
Source: BMC Health Serv Res. 2024 Nov 23;24:1455. doi: 10.1186/s12913-024-11975-0 (PMC11585134; doi:10.1186/s12913-024-11975-0)
Supplement: Supplementary file 3 — Supplementary Material 3. [file 12913_2024_11975_MOESM3_ESM.docx]

| **Supplementary file 3. Barriers to adoption of 3D printing based on the TOE framework** | | |
| --- | --- | --- |
| **Themes** | **Subthemes** | **Quotes** |
| **Organization** (n=33) | Costs (n=7) | “It's the costs. That the printer is expensive, that the material is expensive.”- R1RD |
|  |  | “Cost is another concern, especially if using expensive materials like metals.” - R3RF |
|  |  | “Yes, estimated cost today I should say maybe... Between 60,000-100,000 to get the permits done. So, you have high costs for a small product. Then you might need the CE-mark, also for simple products. I think you are close half a million before you're done.”- R1RA |
|  |  | “The cost issue is not entirely clear. It may be uncertain who is responsible for covering the costs of using 3D printing in medical applications.”- R4RC |
|  |  | “Demonstrating direct cost savings is challenging, as the financial gains are often indirect. For instance, for maxillofacial surgery, the cost of wafers dropped significantly, and turnaround time improved. While this shows tangible benefits, some gains, such as fewer complications, aren't always directly translated into monetary savings.”- R2RG |
|  |  | “Developing and introducing 3D printed products can be a costly process, and it can be difficult to find a profitable business model to cover these costs.”- R2RA |
|  |  | “High costs of 3D printing equipment and materials might be a challenge for healthcare, especially if it requires to order equipment externally from third part or outsource services.”- R1RG |
|  | No central decision at the hospital level (n=6) | “Some doctors have been using AM for many years due to personal interest, but there is no institutional-level decision on AM adoption. It remains personalized, with doctors who are interested using AM, but the hospital as an institution hasn't taken a centralized decision on it."- R4RC |
|  |  | “There is no centralized printing facility. It's mostly local initiatives from different departments."- R6RC |
|  |  | “There seems to be a need for more centralized initiative and interest to drive and promote the use of 3D printing in healthcare i  the region. This can improve communication between authorities, researchers and industry, and facilitate the change process.”- R1RC |
|  |  | “Who introduced it? Doctors, hospitals, or patient demand? So far, it's the doctors. They've been driving this due to their enthusiasm. Decisions are often made at different levels within the decentralized healthcare organization…”- R2RF |
|  |  | “Our funding is now from “Image and function”. I would have rather seen that there was more centralized. Actually, it doesn't matter that much where the money comes from, but still somehow it sends a signal, so to speak, about that it is important.”- R2RG |
|  |  | "However, adopting it requires a significant time investment, often beyond their regular work hours. While our hospital hasn't actively promoted AM adoption, my department, which focuses on medical physics and technology, is attempting to take the lead.” -R2RB,R3RB |
|  | Requirements for premises (n=4) | “It becomes like a process with all the toxic substances that arise, all the gases and work environment problems. That you have to adapt the premises where the printer is located.”- R1RD |
|  |  | “Another challenge was finding premises for us. We still don't have optimal premises for that.”- R2RG |
|  |  | “And then it's just the money, because it will cost money to hire staff, build routines, find premises, equipment, validation, interactivity.”- R2RC |
|  |  | “[I]f we want to acquire new technologies, it demands suitable premises. But first and foremost, that there are premises at all. When we started up our 3D center, we experienced difficulties around practical things like premises.”- R1RB |
|  | Requirements for support (n=4) | “The hospital's record system and IT-support must be developed to enable effective management and prescription of 3D printed drugs.”- R2RC |
|  |  | “In terms of adopting the technology with their own equipment, there emerges a notable support issue. The healthcare sector often lacks adequate IT or MT support staff. This raises concerns about investing in such technology, as the question arises: How would they receive the necessary support? There's even the possibility that they might need to hire additional personnel to manage and maintain the technology."- R1RF,R3RF |
|  |  | "We started by doing some 3D planning with maxillofacial surgeons around 2018. However, I was the only engineer at the clinic, assisting them with everything. The problem was that although we had some resources, such as 3D printers, software, and knowledge in the hospital, we couldn't assist other clinics because I was the only one available."- R1RE |
|  |  | "We're also pondering the need for a support organization and its placement. Should it be centralized or integrated into specific departments?.”- R2RF |
|  | Requirements for technical competence (n=4) | “Segmenting 3D models can be difficult to use and requires training and expertise.”- R1RD |
|  |  | “There's even the possibility that [we] might need to hire additional personnel to manage and maintain the technology.”- R1RF,R3RF |
|  |  | "Another hinder is that you have to know about the technology. Well, if we take this example; someone must physically go to a computer and cold chamber and have a model and spend some time on it. I know there are manufacturers that come up with user-friendly software programs to be able to manage this chain. So it is a bit much for doctors to have both the clinical knowledge but also the technical one perhaps?.”- R2RA |
|  |  | “The 3D printing technology requires special knowledge and skills, which makes it difficult for some companies or hospitals to introduce the technology…”- R4RC |
|  | Unclear need (n=4) | ”I wouldn't say it's widespread. But I might make an average of one printout a month. It's not every month, but sometimes there might be two or three prints in a month. Sometimes it takes a little longer before there are any referrals. it depends on the demand. Clinicians doesn’t always come up with the idea that they can make a printout. If you have a lecture about it or something, there are usually a few referrals after that."- R1RD |
|  |  | “Lack of clarity regarding the actual need for additive manufacturing in healthcare.”- R1RE |
|  |  | “It's easy to say 3D printing for a year or so, it sounds cool but there has to be a clear application area.”- R2RA |
|  |  | “We are far from what I think the need would actually be. That is, if people discovered the benefits of it. I probably have as a goal maybe within three or four years, 500 referrals per year. The referrals might request more complicated prints then.”- R2RG |
|  | Conservative healthcare professionals (n=4) | “Another hindrance is persuading other surgeons to recognize the benefits of using 3D planning. There are some "old-school" surgeons who are skeptical about the effectiveness of 3D planning and prefer to proceed without any planning at all.”- R1RE |
|  |  | "Relying on the accuracy of the guides can be a point of skepticism. Not everyone may have complete faith in them and might prefer to handle procedures themselves.”- R1RF,R3RF |
|  |  | ”There may be some conservatism in healthcare when it comes to embracing new technologies like 3D printing. It could be hard to get people to consider adopting the technology if they can't see clear benefits and cost savings.”- R2RA |
|  |  | “The slow adoption of AM technology in medicine can be attributed to several factors. Firstly, 3D printing represents a significant shift in how medical devices and components are produced, and this disruptive change may cause hesitation among some conservative healthcare professionals."- R4RC |
| **Environment** (n=15) | Complex regulatory framework (n=7) | “When using 3DP in-house, the production of medical devices can meet regulatory requirements that require approvals from the Swedish Medicines Agency and expensive monitored studies. This might be challenging and a costly process for hospital.”- R2RA |
|  |  | "There's a learning curve for everyone as we adapt to the new regulatory framework, the Medical Device Regulation (MDR), while also grappling with financing and resource limitations.”- R1RB, R2RB |
|  |  | “In order to use 3DP in cellular therapies, one must meet rigorous regulations and requirements from both the Swedish Medical Products Agency and the National Board of Health and Welfare”- R1RA |
|  |  | "I've been looking at the regulatory aspect, specifically MDR regulations for these 3D printed medical products. This legal and regulatory aspect is crucial to consider from the outset."- R3RF |
|  |  | "Adoption varies. Some products fit within the adaptable category. Some individualized products are acceptable. As for AI-based products with self-learning capabilities, regulations impose constraints. The intersection of MDR and GDPR creates challenges. The adoption rate is influenced by these complexities.”- R2RF |
|  |  | ”A current barrier is the MDR regulations. The reason behind this is the increased processing requirements.”- R1RE |
|  |  | "Surgical guides and implants, on the other hand, fall under the category of medical devices, which requires stricter manufacturing standards."- R6RC |
|  | Uncertainty about regulations (n=5) | “There are those who work with the technology without taking the regulation into account and may do things that you are not allowed to do. On the other hand, there are users that follow the regulation and unfortunately a few assume that you are not allowed to do anything. The right thing to do is somewhere in between. You can do a lot, but you have to do it the right way.”- R2RB |
|  |  | “Uncertainty about limits and guidelines for 3D printing in health care. Variations in understanding and interpretation of legislation among clinics and practices.“- R3RB |
|  |  | “The legal and regulatory aspect, including MDR-regulation for 3D printed medical products, is complicated. There is uncertainty about how these rules affect the use of the technology.”-R1RF,R2RF,R3RF |
|  |  | “There is uncertainty surrounding the regulatory framework for 3D printing of pharmaceuticals, and it is unclear how this type of manufacturing should be classified (extemporaneous preparation or other type of manufacturing).”- R1RC |
|  |  | “Yes, I mean, MDR has been a mess to deal with because of that. So it hasn't exactly made our job any easier. But I will say this, you have to have somebody who is quite regulatory savvy, and that's hard to find, I'll say. It's easier to find a 3D printing geek, but finding an MDR geek is more difficult, and in my case it's been me who has had to read and learn with MDR. So that it is, I would say, it is a challenge.”- R2RG |
|  | Difficult to interpret regulations (n=3) | “It might be doubts about appropriate methods and approaches, permitted according to current regulations. Security issues around the use of 3D printing and their impact on patient safety might hinder further spread of the technology.”- R1RG |
|  |  | "It's possible, but the regulation side is still a bit unclear. I haven't delved much into the MDR and its implications. In region 7, they printed a PEEK implant for academic plastic surgery under the old regulation, MDD, as it might be more challenging with the MDR in force. So, I'm not sure how well they have  addressed these issues.”- R6RC |
|  |  | “In addition, the interpretation of the law depends on each region. It's not just a law that you learn, but each region interprets it in its own way, which makes it even more difficult. But if region help each other and exchange regulation knowledge under the MDR, I think it would be very valuable. It would be a success.”-R1RF,R2RF,R3RF |
| **Technology** (n=4) | Not applicable for all areas (n=4) | “It is good for seeing skeletons. It's not quite as good at soft parts. Very small vessels are also difficult to get any good 3D printing on. Because it depends on the resolution of the DT device, that the vessels are too small for it to be visible well on the DT.”- R1RD |
|  |  | “For instance, if a surgeon is experienced in fixing a hand fracture, they may not see the need for a 3D plan. But in unique cases, they may not be aware that they can contact us for 3D planning.”- R1RE |
|  |  | “We have encountered cases where patients have tumors in the mandible that require resetting. Previously, they used a single-piece approach, which was simple and fast. However, with 3D planning, it's possible to create guides that involve 2-3 pieces. Nevertheless, this makes the surgery more complex and may add some time to the procedure, which some surgeons may not prefer.”- R1RE |
|  |  | “Some doctors express skepticism about the accuracy of surgical guides produced by 3D printing, raising concerns about patient safety. ”- R1RF, R3RF |
